# Cartilage tympanoplasty for retraction pocket of the tympanic membrane in children

**DOI:** 10.3389/fped.2024.1314184

**Published:** 2024-02-14

**Authors:** Milan Urík, Kateřina Sobotková, Michal Bartoš, Josef Machač, Vít Kruntorád, Jiří Jarkovský

**Affiliations:** ^1^ Department of Pediatric Otorhinolaryngology, University Hospital Brno and Faculty of Medicine of Masaryk University, Brno, Czech Republic; ^2^ Institute of Biostatistics and Analyses, Masaryk University, Brno, Czech Republic

**Keywords:** retraction pocket, tympanic membrane, cartilage tympanoplasty, hearing, children

## Abstract

**Background:**

Retraction pocket (RP) of the tympanic membrane (TM) is a common pathology in children that can cause ossicular chain erosion, cholesteatoma formation, and potentially life-threatening complications of cholesteatoma. This study assessed the functional and anatomical results of cartilage grafting in children with severe RP of the TM.

**Methods:**

This was a retrospective review of 212 children from a tertiary referral center.

**Results:**

We identified significant differences in hearing results, indication criteria, and location of TM fixation between stages II and III of RP (according to Charachon). We observed a significantly higher incidence of RP in boys than in girls.

**Conclusions:**

Cartilage tympanoplasty for retraction pocket of the tympanic membrane in children is a safe procedure with good anatomical and hearing results.

## 1 Introduction

Retraction pocket (RP) of the tympanic membrane is a common pathology, especially in children. It can affect both parts of the tympanic membrane (TM), pars tensa and pars flaccida. Retraction pocket can cause ossicular chain erosion, cholesteatoma formation, and potentially life-threatening complications of cholesteatoma. Several theories describe RP's development. Many authors suggest that negative middle ear pressure is the main cause of this condition (1–3), but we now know that inflammation plays a key role in RP's pathogenesis and its progression to cholesteatoma (4–7).

The three systems most used for classification of retraction pockets are those of Sadé (8), Tos (9), and Charachon (10). These systems grade the severity of RP, but they do not explain the pathogenesis or possibility of retraction pocket's progression.

No consensus exists concerning the optimal treatment of RPs and so different treatment strategies are pursued. Insertion of a ventilation tube is standardly used to prevent RP's progression, but in many cases this treatment is not efficient (11, 12). Some authors prefer a watch and wait strategy. The main surgical procedure for treating RP in children is cartilage tympanoplasty.

The aim of this study was to assess the efficacy of cartilage tympanoplasty for RPs in children in a tertiary referral center.

## 2 Materials and methods

This retrospective study concerned 212 cases of RPs of the TM in children operated in a tertiary referral center from January 2010 to December 2020 by dissection of the RP followed by reinforcement of the TM using a tragal or conchal cartilage graft. Twenty-six of these patients underwent surgery on both sides. The mean patient age (± SD) was 10.2 ± 3.7 years and mean postoperative follow-up time was 65 ± 15 months. Some children had associated diseases or malformations that could account for the dysfunction of the eustachian tube and subsequent development of the tympanic retraction. These included 29 cases of velopalatine clefts, 2 cases of Down syndrome, 2 cases of Pierre-Robin Syndrome, 1 case of DiGeorge syndrome, and 1 case of Apert syndrome.

Retraction pockets were graded according to the RP classification system proposed by Charachon (10). In our opinion, this classification is clinically beneficial. It has been used in our department for a long time.

The classifications are as follow: Stage I, wherein the entire surface of the pocket can be visually controlled and there is no fixation to the middle ear structures; Stage II, wherein the whole surface of the pocket can be visually controlled but there is fixation of the RP to the middle ear structures; and Stage III, wherein part of the pocket is not visible because of its deep extension into the middle ear cavities or because it is filled with abundant squamous debris. Tympanoplasty was performed only in RPs of stages II and III.

Our series concerned 117 Stage II and 95 Stage III pars tensa retraction pockets. In 40 cases there was an associated RP of the pars flaccida in Stage II pars tensa retraction pockets. In 18 cases there was an associated RP of the pars flaccida in Stage III pars tensa retraction pockets. A contralateral middle ear disease was observed in 26 cases.

In all patients, we standardly provided elevation of the RP, excision of the RP, and reconstruction of the tympanic membrane using tragal or conchal cartilage and perichondrium. Perichondrium was applied against the anterior or posterior wall of the external ear canal to stabilize the graft and prevent its secondary displacement.

Postoperatively, patients were regularly monitored by micro-otoscopy and audiometry. Computed tomography (CT) of the temporal bone was not performed. Revision surgery was carried out in cases of tympanic retraction's recurrence, otoscopic suspicion of cholesteatoma, tympanic perforation, poor hearing outcome, or systematically when the RP was disrupted during the first operation. The correlation between the risk of retraction's recurrence and several clinical or therapeutic parameters was statistically analyzed as was correlation between stages of RP and audiological outcomes.

Absolute and relative frequencies were given for categorical variables, mean, standard deviation (SD), median, minimum, and maximum for continuous variables. Data normality was tested using the Shapiro–Wilk test. Due to non-confirmation of normality in the monitored variables, the Mann–Whitney *U*-test was used to evaluate differences between the two groups. In addition, Bonferroni correction was applied when evaluating differences in hearing at individual frequencies. Fisher's exact test was used to evaluate the categorical variables. A binomial test was used to test gender equality in relation to disability. All tests were run at a 5% level of significance. The results were evaluated using IBM® SPSS® Statistics Version 27.

## 3 Results

The study enrolled 212 consecutive patients undergoing cartilage tympanoplasty between 3 and 19 years of age. Median age was 10 years. Characteristics of patients in analyzed groups are shown in Table 1. We treated 89 (42%) girls and 123 (58%) boys, indicating a statistically significant difference in gender representation (*p* = 0.023). Destruction of the ossicles was present in 18 patients (8.5%). In 109 patients (51.4%), only the otomicroscopy findings were indication for surgery. In 103 patients (48.6%), otomicroscopy and audiometry (hearing loss) findings were indication for surgery. In Stage II patients, otoscopy predominated as an indication (57%), while in Stage III patients it was otoscopy + audiometry (59%), (*p* = 0.038).

**Table 1:** Patients characteristics.

Item	*N* (%)
Number of patients	212 (100%)
Sex	
Female	89 (42.0)
Male	123 (58.0)
Age	
Mean	10.2
Median	10
SD	3.7
Min/max	3/19
RP stage	
II	117 (55.2)
III	95 (44.8)
Ossicles	
No	194 (91.5)
Yes	18 (8.5)
Indication	
O	109 (51.4)
O + A	103 (48.6)
Recurrence	
No	199 (93.9)
Yes	13 (6.1)
Surgery	
M	184 (86.8)
M + T	18 (8.5)
Other	10 (4.7)

O, otoscopy; A, audiometry; M, myringoplasty; T, tympanoplasty.

Myringoplasty was performed in 184 patients (86.8%), myringoplasty and ossiculoplasty in 28 (13.2%) patients. Recurrence of the RP occurred in 13 patients (6.1%). The most frequent area of the TM fixation (in 153 or 72.2% of cases) was the incudostapedial joint. We identified a statistically significant difference in area of TM fixation between stages II and III (Table 2). Statistically significant differences were found between stages II and III for fixation sites as *capitulum mallei* (16% vs. 5%, *p *= 0.033), promontorium (20% vs. 48%, *p* < 0.001), and stapedial tendon (4% vs. 20%, *p* < 0.001). The ossicle most affected was the incus, in 18 (8.5%) cases. Hearing results are shown in Tables 3, 4. No statistically significant difference in hearing results between stages II and III was observed either before or after surgery. After Bonferroni correction, a statistically significant difference in hearing was found between stages II and III a half year after surgery at frequency 1,000 Hz. Patients with RP of stage III had poorer hearing half a year after surgery at 1,000 Hz than did patients with stage II (*p* = 0.009). There was no statistically significant difference in hearing results between patients with or without ossicular damage. After Bonferroni correction, statistically significant differences in hearing at frequency 4,000 Hz were found between the categories of patients with and without ossicular damage at all monitored times (*p* = 0.003 before surgery, *p* = 0.005 half a year after surgery, *p* = 0.007 a year after surgery). The presence of ossicles at this frequency indicates poorer hearing. No statistically significant difference was found either between recurrence and stage of RP or between recurrence and damage of the ossicles. No statistically significant difference in age was found by stage of RP, by damage of or no damage to ossicles, or by recurrence.

**Table 2:** Differences between groups of stages II and III retraction pocket.

Stadium	II	III	*p*-value
Number of patients	117	95	
Age			
Mean	10.4	9.9	0.491
Median	10	10	
SD	3.8	3.6	
Min	4	3	
Max	19	18	
Recurrence			
No	102 (95.3%)	76 (96.2%)	1.000
Yes	5 (4.7%)	3 (3.8%)	
Indication			
O	61 (57.0%)	32 (40.5%)	**0** **.** **038**
O + A	46 (43.0%)	47 (59.5%)	
Location of the fixation			
Mc	17 (15.9)	4 (5.1)	**0** **.** **033**
Ipl	21 (19.6)	25 (31.6)	0.085
P	21 (19.6)	38 (48.1)	**<0** **.** **001**
S	11 (10.3)	12 (15.2)	0.370
Sc	6 (5.6)	2 (2.5)	0.470
Mca	0 (0.0)	2 (2.5)	0.179
Ic	0 (0.0)	3 (3.8)	0.075
ST	4 (3.7)	16 (20.3)	**<0** **.** **001**
R	0 (0.0)	0 (0.0)	–
Ossicles			
M-	1 (0.9)	0 (0.0)	1.000
I-	9 (8.4)	9 (11.4)	0.617
S-	0 (0.0)	0 (0.0)	–
Surgery			
MP	95 (88.8)	66 (83.5)	0.385
MP + T	9 (8.4)	9 (11.4)	0.617

O, otoscopy, O + A, otoscopy + audiometry; M, malleus; I, incus; S, stapes; MP, myringoplasty; T, tympanoplasty; Mc, *malleus collum*; IpI, *processus longus incus*; P, promontorium; S, stapes; Sc, scutum; Mca, *malleus capitulum*; Ic- I,; ST, stapedial tendon; R, retrotympanum.

Bold values means the statistically significant values.

**Table 3:** Hearing status according to stage of retraction pocket.

Stage	II					III					*p*-value				
Frequency	250	500	1,000	2,000	4,000	250	500	1,000	2,000	4,000	*p*_1_	*p*_2_	*p*_3_	*p*_4_	*p*_5_
Before surgery															
* N*	100	100	100	100	100	73	73	73	73	73					
* *Mean	26.1	25.3	23.4	19.4	22.3	29.2	27.8	26.5	21.8	24.5					
* *Median	25	20	20	15	20	30	25	25	20	20	0.099	0.082	0.047	0.095	0.051
* *SD	11.1	11.7	11.5	9.3	12.4	12.4	11.3	12.3	10.6	11.4					
* *Min	10	5	5	5	5	10	10	10	5	5					
* *Max	55	70	85	70	75	60	60	65	50	55					
½ year after surgery															
* N*	96	96	96	96	96	78	78	78	78	78					
* *Mean	23.0	22.3	20.8	17.7	21.5	25.4	24.2	23.9	19.6	22.6					
* *Median	20	20	20	15	20	20	20	20	15	20	0.115	0.244	**0**.**009**	0.054	0.170
* *SD	10.6	10.1	10.3	9.5	11.0	10.8	11.1	10.2	8.9	9.9					
* *Min	5	5	10	5	5	10	10	5	5	10					
* *Max	60	65	75	70	70	70	70	55	50	65					
1 year after surgery															
* N*	86	86	86	86	86	70	70	70	70	70					
* *Mean	22.2	21.9	20.2	16.7	20.9	23.9	23.4	21.9	17.6	21.7					
* *Median	20	20	20	15	20	20	20	20	15	20	0.084	0.046	0.130	0.204	0.215
* *SD	12.9	13.7	11.5	12.8	14.1	10.6	9.8	9.9	8.4	10.4					
* *Min	10	5	5	5	5	5	10	5	5	10					
* *Max	105	110	80	110	110	70	70	65	45	70					

Bold values means the statistically significant values.

**Table 4:** Hearing status depending on the presence of the ossicles.

	Ossicles −					Ossicles +					*p*-value				
Frequency	250	500	1,000	2,000	4,000	250	500	1,000	2,000	4,000	*p*_1_	*p*_2_	*p*_3_	*p*_4_	*p*_5_
Before surgery															
*N*	16	16	16	16	16	183	183	183	183	183					
Mean	30.9	30.0	27.8	23.4	30.0	26.7	25.8	24.1	20.0	22.3					
Median	30	30	25	20	30	25	25	20	20	20	0.125	0.084	0.134	0.051	**0**.**003**
SD	12.4	12.0	12.5	9.1	11.3	11.9	12.0	12.1	10.4	12.6					
Min	10	10	15	10	15	10	5	5	5	5					
Max	55	60	65	45	50	75	80	85	75	95					
½ year after surgery															
*N*	17	17	17	17	17	180	180	180	180	180					
Mean	25.3	21.8	22.4	20.6	26.5	23.9	23.4	22.3	18.3	21.3					
Median	25	20	20	20	25	20	20	20	15	20	0.155	0.844	0.610	0.070	**0**.**005**
SD	6.7	6.1	7.3	7.9	8.4	11.3	11.3	10.8	9.4	10.7					
Min	15	10	15	10	15	5	5	5	5	5					
Max	35	30	35	45	40	70	70	75	70	70					
1 year after surgery															
*N*	16	16	16	16	16	161	161	161	161	161					
Mean	24.4	24.1	22.2	18.4	25.6	23.0	22.4	20.7	17.0	20.6					
Median	23	20	18	15	20	20	20	20	15	20	0.298	0.300	0.624	0.713	**0**.**007**
SD	9.3	10.4	10.8	10.1	9.8	12.2	12.0	10.4	10.7	12.2					
Min	10	5	5	5	15	5	5	5	5	5					
Max	45	45	45	40	50	105	110	80	110	110					

Bold values means the statistically significant values.

## 4 Discussion

Within a well-defined population of children, we analyzed the indications, complications, as well as anatomical and hearing results in cartilage tympanoplasty for pars tensa retraction pocket (RP) of the tympanic membrane (TM).

Severity of the RP can be assessed by evaluating many different parameters, but commonly including extension of the RP, visibility of the whole area of the RP, presence of keratin debris, possibility to elevate the RP, erosion of the bony structures, and hearing levels (13, 14). Several classification systems exist based upon the evaluation of these parameters. In our opinion, the classification of RP in accordance with Charachon is very useful for deciding about surgical treatment. Stage III is always indicated for tympanoplasty, because there is fixation to the middle ear structures and part of the pocket is not visible due to its deep extension into the middle ear cavity or because it is filled with abundant squamous debris. Stage I does not require a surgical procedure, and strategies of watchful waiting or insertion of ventilation tubes predominate. There is uncertainty as to the optimal treatment in cases of Stage II, wherein the whole surface of the pocket can be visually controlled but there is a fixation of the RP to the middle ear structures. The main reason for tympanoplasty in Stage II is conductive hearing loss, because fixation of the RP to the ossicles is related to damage of the incudostapedial joint or other parts of the ossicular system. In our opinion, precise otomicroscopy is crucial when an RP is at this stage. We recommended surgical treatment in most cases of Stage II because we have shown pathological abnormalities in structure at Stage II leading to cholesteatoma formation (4, 5). Some studies recommend CT scan of the temporal bone in cases of RPs that are not completely visualized (15). Based on our experience, this is not necessary, because surgery is always indicated in these cases. If we suspect cholesteatoma in the area of a deep retraction pocket, we do perform CT.

Pure tone audiometry may be entirely normal in the early stages but progress to conductive hearing loss. Tympanometry can show negative middle-ear pressure due to eustachian tube dysfunction. The central problem at diagnosis is to distinguish stable from progressive disease. In children, the disease tends to pursue a more aggressive course compared to in adults, thus requiring frequent follow-up to allow progression to be identified (15).

Reinforcement tympanoplasty has a place in RP treatment, but this is best reserved for adults with chronic eustachian tube dysfunction (16, 17). Although some authors claim that reinforcement tympanoplasty is not generally performed in children because eustachian tube dysfunction tends to improve with time (15), we do not agree with this statement and our results confirm that tympanoplasty is a reliable solution even in children.

## 5 Conclusions

We identified significant differences in hearing results between stages II and III of RP (according to Charachon staging) indication criteria and location of TM fixation. We observed a significantly higher incidence of RP in boys than in girls. We observed no significant difference between stages II and III in recurrence of the RP. We conclude that cartilage tympanoplasty for retraction pocket of the tympanic membrane in children is a safe technique that prevents recurrences of retraction pockets of the pars tensa and enables hearing improvement whatever the status of the ossicular chain. In cases of damage to the ossicular chain, that chain can be repaired using cartilage graft placed directly into the incudostapedial joint or head of the stapes.

## Data Availability

The raw data supporting the conclusions of this article will be made available by the authors, without undue reservation.
